# Overexpression of microRNA‐138 alleviates human coronary artery endothelial cell injury and inflammatory response by inhibiting the PI3K/Akt/eNOS pathway

**DOI:** 10.1111/jcmm.13074

**Published:** 2017-03-31

**Authors:** Jing‐Bo Li, Hai‐Yang Wang, Ye Yao, Qing‐Feng Sun, Zong‐Hong Liu, Si‐Qi Liu, Jun‐Li Zhuang, Yun‐Peng Wang, Hong‐Yu Liu

**Affiliations:** ^1^ Department of Cardiac Surgery The First Affiliated Hospital of Harbin Medical University Harbin China; ^2^ Department of Vascular Surgery The First Affiliated Hospital of Harbin Medical University Harbin China

**Keywords:** microRNA‐138, PI3K/Akt/eNOS signalling pathway, human coronary artery endothelial cells, inflammatory response

## Abstract

This study aimed to investigate the role of miR‐138 in human coronary artery endothelial cell (HCAEC) injury and inflammatory response and the involvement of the PI3K/Akt/eNOS signalling pathway. Oxidized low‐density lipoprotein (OX‐LDL)‐induced HCAEC injury models were established and assigned to blank, miR‐138 mimic, miR‐138 inhibitor, LY294002 (an inhibitor of the PI3K/Akt/eNOS pathway), miR‐138 inhibitor + LY294002 and negative control (NC) groups. qRT‐PCR and Western blotting were performed to detect the miR‐138, PI3K, Akt and eNOS levels and the protein expressions of PI3K, Akt, eNOS, p‐Akt, p‐eNOS, Bcl‐2, Bax and caspase‐3. ELISAs were employed to measure the expressions of TNF‐α, IL‐4, IL‐6, IL‐8, IL‐10 and nitric oxide (NO) and the activities of lactate dehydrogenase (LDH) and eNOS. MTT and flow cytometry were performed to assess the proliferation and apoptosis of HCAECs. Compared to the blank group, PI3K, Akt and eNOS were down‐regulated in the miR‐138 mimic and LY294002 groups but were up‐regulated in the miR‐138 inhibitor group. The miR‐138 mimic and LY294002 groups showed decreased concentrations of TNF‐α, IL‐6, IL‐8 and NO and reduced activities of LDH and eNOS, while opposite trends were observed in the miR‐138 inhibitor group. The concentrations of IL‐4 and IL‐10 increased in the miR‐138 mimic and LY294002 groups but decreased in the miR‐138 inhibitor group. The miR‐138 mimic and LY294002 groups had significantly decreased cell proliferation and increased cell apoptosis compared to the blank group. These findings indicate that up‐regulation of miR‐138 alleviates HCAEC injury and inflammatory response by inhibiting the PI3K/Akt/eNOS signalling pathway.

## Introduction

Endothelial cells, a single layer of cells that line the inner surface of blood vessels in the human body, inhibit activation of pro‐inflammatory and clotting factors in vessels 1. The endothelial cells are involved in a variety of normal physiological functions, such as controlling vasomotor tone, maintaining blood fluidity, forming new blood vessels, regulating permeability and trafficking cells 2. The loss of endothelial integrity results in several vascular complications, and endothelial cell injury and proliferative dysfunction have been shown to initiate the development of atherosclerosis 1. Furthermore, chronic vascular inflammation due to a persistent increase in circulating inflammatory cytokines is associated with endothelial dysfunction, which results in the development of atherosclerosis and cardiovascular disease 3. Therefore, it is important to identify the cellular and molecular mechanisms underlying endothelial cell injury and inflammatory response to develop new biomarkers and novel therapeutic strategies for endothelial cell injury.

MicroRNAs (miRNAs) are a class of non‐coding RNAs that can result in mRNA degradation or suppress protein translation by binding to the 3′‐untranslated region of their target mRNAs 4. By modulating the expression of target genes, miRNAs can influence different biological processes, such as the cell cycle, proliferation, differentiation, survival and apoptosis 5, 6. Recent studies have reported that miRNAs regulate endothelial cell apoptosis and senescence‐related pro‐inflammatory status 7, 8. MiR‐138 is an important member of miRNA family and has been shown to be a tumour suppressor in many studies 9, 10. Interestingly, miR‐138 was reported to contribute to endothelial cell dysfunction when induced by pro‐inflammatory cytokines 3. Additionally, the biological activities of endothelial cells, such as HCAECs, are associated with the phosphatidylinositol 3‐kinase/protein kinase B/endothelial NO synthase (PI3K/Akt/eNOS) signalling pathway 11, 12, 13. Ou *et al*. 14 reported that the PI3K/Akt/eNOS/NO pathway plays a role in OX‐LDL‐induced endothelial apoptosis. Additionally, Ngalame *et al*. 15 showed that miR‐138 is correlated with the expression of the PI3K/AKT signalling pathway, which controls cell proliferation and apoptosis. Thus, this study aimed to explore the role of miR‐138 and the PI3K/Akt/eNOS signalling pathway in injury of HCAECs and inflammatory response.

## Materials and methods

### Revivification, culture and passage of HCAECs

HCAECs were obtained from Cell Applications, Inc. (San Diego, CA, USA). Liquid nitrogen‐preserved cells were thawed in a 37°C water bath, and the cells were then transferred into 5 ml 10% modified serum‐free cell freezing medium (RPMI) 1640 (Thermo Fisher Scientific Chemical and Biological Products Co., Ltd., Beijing, China). Then, the cells were centrifuged at 1000 r.p.m. for 5 min., and the supernatant was aspirated. After resuspension in 5 ml 10% modified RPMI 1640, the cells were cultured in a culture bottle (culture conditions: 5% CO_2_ atmosphere at 37°C with 95% humidity), and foaming was avoided. After approximately 90% of cells adhered to the wall, subculturing was conducted. The culture solution was aspirated, and the cells were rinsed with phosphate‐buffered saline (PBS) twice and digested with pancreatic enzymes. As the intercellular gap increased, the enzyme solution was aspirated. The cells were resuspended again in the same culture medium mentioned above and routinely subcultured. Passaged cells in exponential growth were selected for further experiments. Injured HCAECs were selected for the construction of HCAEC injury models.

### Establishment of oxidized low‐density lipoprotein‐induced HCAEC injury models

The subcultured HCAECs were centrifuged at 1000 r.p.m. for 5 min., and the supernatant was aspirated. Cells were maintained in RPMI 1640 (containing 100 mol/l OX‐LDL) for 45 min. to establish OX‐LDL‐induced HCAEC injury models. Then, the cells were cultured in normal medium, and an optical microscope was used to observe and compare the morphological changes between the injured cells and normal cells. A LDH kit (Nanjing Jiancheng Bioengineering Institute, Jiangsu, China) was employed to detect LDH activity in the cells.

### Quantitative real‐time polymerase chain reaction (qRT‐PCR)

After the HCAECs were cultured, the medium was aspirated, and HCAECs were washed with PBS twice. Using the TRIzol reagent (Invitrogen, Carlsbad, CA, USA) instructions, total RNA was extracted using the TRIzol one‐step extraction method. Diethylpyrocarbonate (DEPC)‐treated ultrapure water was used to dissolve RNA, and a ND‐1000 UV‐Visible spectrophotometer (NanoDrop Technologies, Wilmington, DE, USA) was used to measure absorbance at 260 nm and 280 nm to determine the RNA purity and concentration. The RNA concentration was adjusted for qRT‐PCR. TaqMan probes (Table 1) were selected, and the reaction was performed in accordance with the kit instructions (Fermentas Inc., Hanover, MD, USA). The reaction conditions were as follows: pre‐denaturation at 95°C for 30 sec., 40 cycles of denaturation at 95°C for 10 sec., annealing at 60°C for 20 sec. and extension at 70°C. With β‐actin as an internal reference, the gene expression was detected by real‐time PCR (ABI Company, Oyster Bay, NY, USA), and gene expression data were calculated with the 2^−ΔΔCt^ method. All experiments were conducted three times.

**Table 1:** The primer sequences used for quantitative real‐time polymerase chain reaction

Target gene	Primer sequence
MiR‐138	F: 5′‐GGTGTCCGTGGAGTCGGCAA‐3′
	R: 5′‐AACTTCACAACACCAGCTTA‐3′
PI3K	F: 5′‐CTTGCCTCCATTCACCACCTCT‐3′
	R: 5′‐GCCTCTAATCTTCTCCCTCTCCTTC‐3′
Akt	F: 5′‐TGTCTCGTGAGCGCGTGTTTT‐3′
	R: 5′‐CCGTTATCTTGATGTGCCCGTC‐3′
eNOS	F: 5′‐CCAGCTAGCCAAAGTCACCAT‐3′
	R: 5′‐GTCTCG GAGCCATACAGGATT‐3′
β‐actin	F: 5′‐CACCACACCTTCTACAATGAGC‐3′
	R: 5′‐GTGATCTCCTTCTGCATCCTGT‐3′

Note: miR‐138, microRNA‐138; PI3K, phosphatidylinositol 3‐kinase; Akt, protein kinase B; eNOS, endothelial nitric oxide synthase.

### Plasmid construction, grouping and HCAEC transfection

Based on the known miR‐138 sequence from National Center for Biotechnology Information (NCBI), along with shuttle vector pcDNA3.1 (Shanghai GenePharma Co., Ltd., Shanghai, China), negative control, miR‐138 mimic and miR‐138 inhibitor plasmids were constructed by Sangon Biotech Co., Ltd., (Shanghai, China). The sequences of miR‐138 mimic, negative control and miR‐138 inhibitor were 5′‐CCUGCUUGCUCAAAUCAAUTT‐3′, 5′‐CAGUACUUUUGUGUAGUACAA‐3′ and 5′‐GGCAUUCACCG CGUGCCUUA‐3′, respectively. The cells (second passage) were digested with pancreatic enzymes and inoculated into 24‐well plates, and the cells grew in a monolayer. The medium was aspirated, and the cells were assigned to five groups: the blank group (transfected with empty vector), the miR‐138 mimic group (transfected with miR‐138 mimic plasmid), the miR‐138 inhibitor group (transfected with miR‐138 inhibitor plasmid), the LY294002 group (treated with the inhibitor LY294002), the miR‐138 inhibitor + LY294002 group (transfected with miR‐138 inhibitor plasmid and treated with LY294002) and the NC group (transfected with negative control plasmid). The transfection procedures were as follows: Plasmid suspension solution (100 μl) was added to the corresponding plates with the injured cells in 200 μl serum‐free Dulbecco's modified Eagle's medium (DMEM) (Gibco Company, Grand Island, NY, USA). After a 3‐h culture, 1 ml DMEM containing 10% serum (HyClone Company, Logan, UT, USA) was added to the cells. In the LY294002 group, the injured cells were treated with LY294002, and in the miR‐138 inhibitor + LY294002 group, the injured cells were transfected with miR‐138 inhibitor and treated with LY294002.

### Western blotting

After a 48‐h transfection, the cells were collected. Then, 1× sodium dodecyl sulphate (SDS) lysis buffer (Beyotime Biotechnology Co., Shanghai, China) was added, and protein extracts were heated at 100°C for 5 min. Proteins (20 μl) were separated with 10% polyacrylamide gel electrophoresis and then transferred onto a polyvinylidene fluoride (PVDF) membrane for 3.5 hrs with a voltage of 48 V. The membrane was sealed in 5% bovine serum albumin (BSA) for 2 hrs at room temperature and was rinsed with 1× Tris‐buffered saline plus Tween (TBST). The primary antibodies (PI3K, Akt, eNOS, p‐Akt, p‐eNOS, Bcl‐2, Bax and caspase‐3) (Cell Signaling Technologies (CST), Beverly, MA, USA; diluted 1: 500) were separately added, and the membrane reacted at 4°C overnight. β‐Actin monoclonal antibody was used as an internal reference. The membrane was washed with 1× TBST and was incubated with the peroxidase‐labelled anti‐rabbit secondary antibody (1: 2000; Santa Cruz Biotechnology, Inc., Santa Cruz, CA, USA) for 1 hr at room temperature. After being washed with TBST, the membrane was incubated with Chemiluminescent Reagent (Thermo Fisher Scientific, Bremen, Germany) for 1 min. and developed by electrochemiluminescence (ECL). Photographs were taken with a gel imaging system (Bio‐Rad, Inc., Hercules, CA, USA). Optical density (OD) analysis of all Western blotting bands was performed. The (OD value of target band)/(OD value of internal reference band) was regarded as the relative expression of protein.

### Enzyme‐linked immunosorbent assay (ELISA)

According to the ELISA kit (R&D Systems, Minneapolis, MN, USA) instructions, the concentrations of TNF‐α, IL‐4, IL‐6, IL‐8 and IL‐10 were detected after 48 hrs of transfection. Coating buffer was used to dilute the antibody to 1 μg/ml, and the diluted antibody was then added to 96‐well plates (100 μl/well) and cultured at 4°C overnight. Afterwards, the coating buffer was aspirated and the plate was washed three times. Each well was incubated with blocking buffer (150 μl) at room temperature for 1 hr. Then, the plate was washed again, followed by addition of 100 μl serum samples or standards. With the blank control set, the plate was cultured for 2 hr at room temperature. After being rinsed, each well was incubated for 1 hr at room temperature after addition of 100 μl horseradish peroxidase (HRP)‐labelled avidin. The wells were rinsed again, and then 100 μl tetramethylbenzidine (TMB) substrate was added to each well for a 15 min. reaction at room temperature, which was terminated by 2 M sulphuric acid (50 μl/well). The absorbance of each well was measured at a wavelength of 450 nm, and the standard curve was generated to determine the concentrations of TNF‐α, IL‐4, IL‐6, IL‐8 and IL‐10 (ng/ml).

### Detections of NO concentration and endothelial nitric oxide synthase (eNOS) activity

The NO concentration was detected using the following protocol. After a 48‐h transfection, the cell supernatant was collected and then centrifuged at 3000 r.p.m. for 20 min. for analysis of the NO concentration following the NO kit instructions (Nanjing Jiancheng Bioengineering Institute). The sample and mixing reagents (R1 and R2) were combined and incubated in a water bath for 60 min. at 37°C, followed by the addition of R3 and R4. The mixture was incubated for 40 min. at room temperature and centrifuged at 3500 r.p.m. for 10 min. The supernatant was collected, mixed with colorimetric reagent and allowed to react for 10 min. prior to analysis of the OD_550_. The eNOS activity was determined by the following processes. After 48 hrs of transfection, the cells were collected to detect the eNOS activity according to NOS kit instructions (Nanjing Jiancheng Bioengineering Institute). The cells were disrupted using PBS and centrifuged to obtain supernatant for the later use. According to the instructions, R1 and R2 were added and reacted for 5 min. at 37°C, followed by the addition of R4 and R5. The OD_530_ value was measured.

### MTT assay

When the cell density reached 80%, the cells were rinsed with PBS twice and digested with 0.25% pancreatic enzymes to obtain single cell suspensions. After being counted, the cells were inoculated into 96‐well plates (3 × 10^3^–6 × 10^3^ cells/well; 200 μl/well). Each group was processed as described above, with three replicate wells. The cells were cultured in a 5% CO_2_ atmosphere at 37°C, and 20 μl 5 mg/ml 3‐(4,5)‐dimethylthiahiazo(z‐y1)‐3,5‐di‐phenytetrazoliumromide (MTT) (Sigma‐Aldrich Chemical Company, St Louis, MO, USA) was added to the cells for 4 hrs. Then, the supernatant was aspirated, and 150 μl dimethylsulphoxide (DMSO) was added to each well, followed by 10 min. of low‐speed shaking. At 24, 48 and 72 hrs, the OD value was detected at 490 nm. With time as the *X*‐axis and OD value as the *Y*‐axis, the cell activity curve was drawn.

### Flow cytometry

After being processed for 48 hrs, the cells in each group were digested by ethylenediaminetetraacetic acid (EDTA)‐free pancreatic enzymes. Then, the cells were centrifuged at 1000 r.p.m. for 5 min. with the supernatant aspirated. After washing with cold PBS three times, the cells were again centrifuged with the supernatant aspirated. In accordance with the Annexin‐V‐FITC apoptosis kit (Sigma‐Aldrich Chemical Company) instructions, 150 μl binding buffer and 5 μl Annexin‐V‐FITC were added to each tube and fully mixed. Then, the cells were cultured for 15 min. at room temperature in the dark. After that, 100 μl Binding buffer and 5 μl propidium iodide (PI) (Sigma‐Aldrich Chemical Company) were added and fully mixed. Finally, cell apoptosis was detected by flow cytometry.

### Statistical analysis

Statistical Analysis Commercial software was used for statistical analysis (SPSS 21.0; SPSS Inc., Chicago, IL, USA). Data are expressed as the mean ± standard deviation (S.D.). Comparisons between two groups were performed using *t*‐tests and those among more than two groups using one‐way analysis of variance (anova). Pairwise comparison was performed by LSD *t*‐tests. *P* < 0.05 indicates significant differences.

## Results

### Analysis of HCAEC morphology and LDH activity for the selection of HCAEC injury models

**Figure 1:**
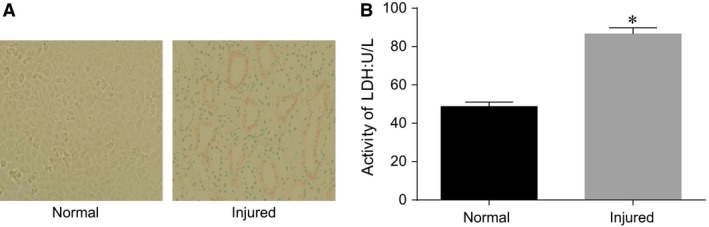
Analysis of HCAEC morphology and LDH activity for the selection of HCAEC injury models. 
Note: **A**, cell morphology of the normal HCAECs and injured HCAECs under a microscope; **B**, LDH activities of the normal HCAECs and injured HCAECs; *P < 0.05 compared with the normal cells; HCAECs, human coronary artery endothelial cells; LDH, lactate dehydrogenase.

Under a microscope, the cells in the control group showed normal morphology, with a cobblestone‐like appearance (Fig. 1A). The injured HCAECs exhibited relatively uniform cellular morphology. However, compared with the normal HCAECs, the injured HCAECs were abnormally elongated and loosely connected with each other, showing irregular cobblestone‐like morphology. In addition, compared to the normal HCAECs, the LDH activity was significantly increased in the injured HCAECs (*P* < 0.05; Fig. 1B). Based on the cellular morphology and LDH activity, the HCAEC injury models were established successfully.

### Comparisons of miR‐138, PI3K, Akt and eNOS expressions between the normal HCAECs and HCAEC injury models

**Figure 2:**
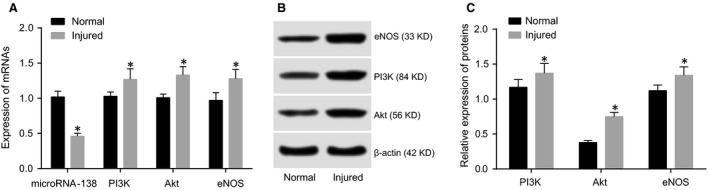
Comparisons of miR‐138, PI3K, Akt and eNOS expressions between the normal HCAECs and injured HCAECs. 
Note: **A**, qRT‐PCR was used to detect the expression of miR‐138 and the mRNA expressions of PI3K, Akt and eNOS in the normal HCAECs and injured HCAECs; **B**, Western blotting was used to detect the protein expressions of PI3K, Akt and eNOS in the normal HCAECs and injured HCAECs; **C**, PI3K, Akt and eNOS protein expressions; *P < 0.05 compared with the normal HCAECs; HCAECs, human coronary artery endothelial cells; qRT‐PCR, quantitative real‐time polymerase chain reaction; PI3K, phosphatidylinositol 3‐kinase; Akt, protein kinase B; eNOS, endothelial nitric oxide synthase.

The mRNA expressions of miR‐138, PI3K, Akt and eNOS in the HCAEC injury models were detected by qRT‐PCR (Fig. 2A). Compared with the normal HCAECs, miR‐138 expression was significantly down‐regulated, while PI3K, Akt and eNOS mRNA expressions were significantly up‐regulated in the HCAEC injury models (all *P* < 0.05). According to the Western blot analyses, PI3K, Akt and eNOS protein expressions were strongly up‐regulated in the HCAEC injury models (all *P* < 0.05, Fig. 2B and C), indicating that down‐regulation of miR‐138 expression activated the PI3K/Akt/eNOS signalling pathway in injured HCAECs. Negative regulation of the PI3K/Akt/eNOS signalling pathway by miR‐138 was observed.

### Comparisons of miR‐138, PI3K, Akt and eNOS expressions among groups after transfection

**Figure 3:**
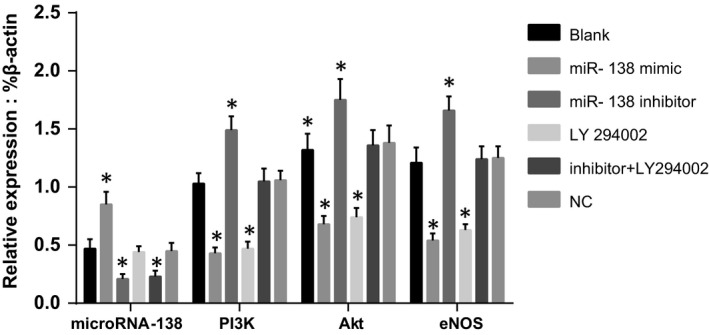
Comparisons of miR‐138, PI3K, Akt and eNOS mRNA expressions among five groups after transfection. 
Note: *P < 0.05 compared with the blank group; qRT‐PCR, quantitative real‐time polymerase chain reaction; PI3K, phosphatidylinositol 3‐kinase; Akt, protein kinase B; eNOS, endothelial nitric oxide synthase.

The mRNA expressions of miR‐138, PI3K, Akt and eNOS in the groups after transfection were detected by qRT‐PCR and are shown in Figure 3. The blank group had low miR‐138 expression. Compared with the blank group, miR‐138 expression was significantly up‐regulated in the miR‐138 mimic group but was strongly down‐regulated in the miR‐138 inhibitor and miR‐138 inhibitor + LY294002 groups (all *P* < 0.05). However, there were no significant differences in miR‐138 expression in the LY294002 and NC groups compared to the blank group (both *P >* 0.05). The blank group showed high expression of PI3K, Akt and eNOS mRNA. Compared with the blank group, PI3K, Akt and eNOS mRNA expressions were up‐regulated in the miR‐138 inhibitor group but were down‐regulated in the miR‐138 mimic and LY294002 groups (all *P* < 0.05), and there was no significant difference between the miR‐138 mimic and LY294002 groups (*P >* 0.05). There were no significant differences in PI3K, Akt and eNOS mRNA expressions among the blank, NC and miR‐138 inhibitor + LY294002 groups (all *P >* 0.05).

### Comparisons of PI3K, Akt and eNOS protein expressions among five groups after transfection

**Figure 4:**
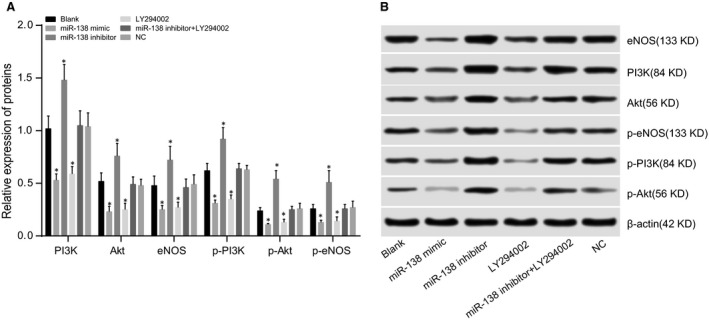
Comparisons of PI3K, Akt and eNOS protein expressions among five groups after transfection. 
Note: **A**, relative expressions of PI3K, Akt, eNOS, p‐PI3K, p‐Akt and p‐eNOS proteins; *P < 0.05 compared with the blank group; **B**, protein expressions of PI3K, Akt, eNOS, p‐PI3K, p‐Akt and p‐eNOS detected by Western blotting; PI3K, phosphatidylinositol 3‐kinase; Akt, protein kinase B; eNOS, endothelial nitric oxide synthase.

The protein expressions of PI3K, Akt and eNOS in the groups after transfection were detected by Western blotting and are shown in Figure 4. PI3K, Akt, eNOS, p‐PI3K, p‐Akt and p‐eNOS proteins were highly expressed in the blank group. Compared to the blank group, PI3K, Akt, eNOS, p‐PI3K, p‐Akt and p‐eNOS protein expressions were significantly up‐regulated in the miR‐138 inhibitor group (all *P* < 0.05) but were substantially down‐regulated in the miR‐138 mimic and LY294002 groups (all *P* < 0.05), while there were no significant differences in these protein expressions between the miR‐138 mimic and LY294002 groups (all *P >* 0.05). There were no significant differences in PI3K, Akt, eNOS, p‐PI3K, p‐Akt and p‐eNOS protein expressions among the blank, NC and miR‐138 inhibitor + LY294002 groups (all *P >* 0.05).

### Comparisons of TNF‐α, IL‐4, IL‐6, IL‐8 and IL‐10 concentrations and LDH activity among five groups after transfection

**Figure 5:**
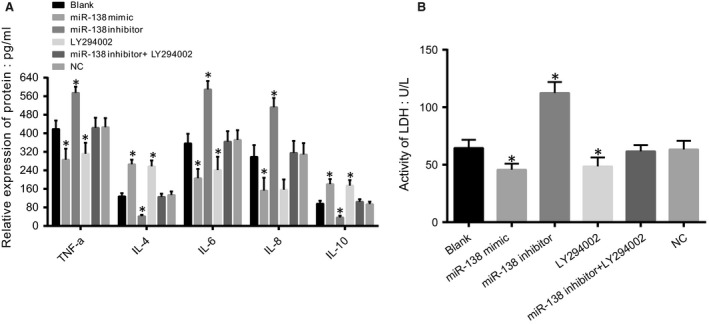
Comparisons of TNF‐α, IL‐4, IL‐6, IL‐8 and IL‐10 and LDH activity among five groups after transfection. 
Note: **A**, diagram of TNF‐α, IL‐4, IL‐6, IL‐8 and IL‐10 concentrations detected by ELISA;** B**, detection of LDH activity; *P < 0.05 compared with the blank group; ELISA, enzyme‐linked immunosorbent assay; LDH, lactate dehydrogenase; TNF‐α, tumour necrosis factor‐α; IL‐4, interleukin 4; IL‐6, interleukin 6; IL‐8, interleukin 8; IL‐10, interleukin 10.

ELISAs were performed to detect the concentrations of TNF‐α, IL‐4, IL‐6, IL‐8 and IL‐10 in HCAECs, and the results are shown in Figure 5A. Compared to the blank group, the concentrations of TNF‐α, IL‐6 and IL‐8 significantly decreased in the miR‐138 mimic and LY294002 groups, but the opposite trend was noted in the miR‐138 inhibitor group (all *P* < 0.05). IL‐4 and IL‐10 concentrations significantly decreased in the miR‐138 inhibitor group, but opposite trends were observed in the miR‐138 mimic and LY294002 groups compared to those in the blank group (both *P* < 0.05). There were no significant differences in the concentrations of TNF‐α, IL‐4, IL‐6, IL‐8 and IL‐10 between the miR‐138 mimic and LY294002 groups and among the blank, NC and miR‐138 inhibitor + LY294002 groups (all *P >* 0.05).

The LDH activity assays (Fig. 5B) showed that compared to the blank group, LDH activity was significantly increased in the miR‐138 inhibitor group but was strongly decreased in the miR‐138 mimic and LY294002 groups (all *P* < 0.05). There were no significant differences in the LDH activity between the miR‐138 mimic and LY294002 groups and among the blank, NC and miR‐138 inhibitor + LY294002 groups (all *P >* 0.05).

### Comparisons of NO concentration and eNOS activity among five groups after transfection

The NO concentration analysis indicated that the NO concentration significantly increased in the miR‐138 inhibitor group but decreased in the miR‐138 mimic and LY294002 groups compared to that of the blank group (all *P* < 0.05). No significant differences in NO concentration were observed between the miR‐138 mimic and LY294002 groups and among the blank, NC and miR‐138 inhibitor + LY294002 groups (all *P >* 0.05).

According to the eNOS activity assays, compared with the blank group, eNOS activity significantly increased in the miR‐138 inhibitor group, but an opposite trend was observed in the miR‐138 mimic and LY294002 groups (all *P* < 0.05). There were no significant differences in the eNOS activity between the miR‐138 mimic and LY294002 groups and among the blank, NC and miR‐138 inhibitor + LY294002 groups (all *P >* 0.05; Table 2).

**Table 2:** Comparisons of NO concentration and eNOS activity among five groups after transfection

Group	NO (μmol/l)	eNOS activity (pmol/min/mg)
Blank	58.8 ± 1.12	16.21 ± 0.39
MiR‐138 mimic	36.07 ± 1.09*	9.77 ± 0.42*
MiR‐138 inhibitor	68.5 ± 2.27*	20.00 ± 0.40*
LY294002	35.81 ± 0.97*	8.98 ± 0.38*
MiR‐138 inhibitor + LY294002	56.34 ± 1.01	15.66 ± 0.24
NC	59.2 ± 1.20	15.90 ± 0.46

Note: miR‐138, microRNA‐138; NO, nitric oxide; eNOS, endothelial nitric oxide synthase; NC, the negative control group; *P < 0.05 compared with the blank group.

### Comparisons of HCAEC proliferation among five groups after transfection

**Figure 6:**
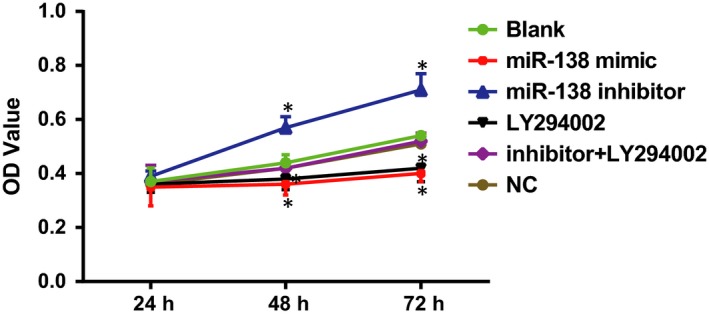
Comparisons of HCAEC proliferation among five groups after transfection. 
Note: *P < 0.05 compared with the blank group; HCAECs, human coronary artery endothelial cells.

The MTT assay indicated that no significant differences in cell proliferation were found among the groups at 24 hrs (all *P* > 0.05; Fig. 6). Additionally, there was no significant difference in OD values among the blank, NC and miR‐138 inhibitor + LY294002 groups at three time‐points (all *P* > 0.05). At 48 hrs and 72 hrs, compared with the blank group, cell proliferation was significantly decreased in the miR‐138 mimic and LY294002 groups, and the OD values were significantly different (all *P* < 0.05). No significant difference in OD values was observed between the miR‐138 mimic and LY294002 groups (*P* > 0.05). Compared to the blank group, the cell proliferation capacity in the miR‐138 inhibitor group significantly increased, and OD values showed significant difference (*P* < 0.05).

### Comparisons of HCAEC apoptosis rate among five groups after transfection

**Figure 7:**
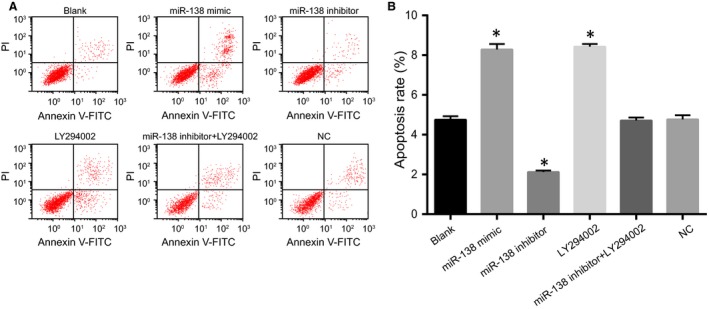
Comparisons of HCAEC apoptosis rate among five groups after transfection. 
Note: **A**, flow cytometry of HCAEC apoptosis detected by the Annexin V/PI method; **B**, diagram of HCAEC apoptosis rate; *P < 0.05 compared with the blank group; HCAECs, human coronary artery endothelial cells.

The cell apoptosis rates in the blank, miR‐138 mimic, miR‐138 inhibitor, LY294002, miR‐138 inhibitor + LY294002 and NC groups were 4.75%, 8.27%, 2.16%, 8.43%, 4.71% and 4.77%, respectively. Compared with the blank group, the cell apoptosis rates significantly decreased in the miR‐138 inhibitor group but increased in the miR‐138 mimic and LY294002 groups (all *P* < 0.05). There was no significant difference in the cell apoptosis rate between the miR‐138 mimic and LY294002 groups and among the blank, NC and miR‐138 inhibitor + LY294002 groups (all *P >* 0.05; Fig. 7).

### Comparisons of apoptosis‐related protein expressions among five groups after transfection

**Figure 8:**
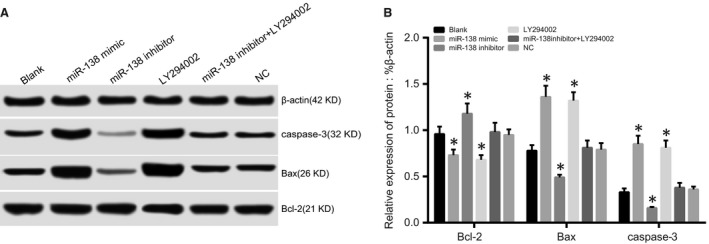
Comparisons of apoptosis‐related protein expressions among five groups after transfection. 
Note: **A**, Bax, cleaved caspase‐3 and Bcl‐2 protein expressions detected by Western blotting; **B**, Bax, cleaved caspase‐3 and Bcl‐2 protein expressions; *P < 0.05 compared with the blank group.

After 48 hrs of transfection and treatment, no significant differences in apoptosis‐related protein expressions were found among the blank, NC and miR‐138 inhibitor + LY294002 groups (all *P* > 0.05). Compared to the blank group, Bax and caspase‐3 proteins were significantly up‐regulated, but Bcl‐2 protein was significantly down‐regulated in the miR‐138 mimic and LY294002 groups (all *P* < 0.05). There was no significant difference in apoptosis‐related protein expressions between the miR‐138 mimic and LY294002 groups (*P* > 0.05). Compared with the blank group, Bax and caspase‐3 proteins were significantly down‐regulated, whereas Bcl‐2 protein was significantly up‐regulated in the miR‐138 inhibitor group (all *P* < 0.05; Fig. 8).

## Discussion

This study explored the roles of miR‐138 and the PI3K/Akt/eNOS signalling pathway in HCAEC injury and inflammatory response. Our data showed that up‐regulation of miR‐138 alleviates HCAEC injury and inflammatory response by inhibiting the PI3K/Akt/eNOS signalling pathway.

We found that compared with the normal cells, miR‐138 expression was significantly down‐regulated, while PI3K, Akt and eNOS mRNA expressions were strongly up‐regulated in the HCAEC injury models. These results indicated that down‐regulation of miR‐138 expression activated the PI3K/Akt/eNOS signalling pathway in the injured HCAECs. Patients with coronary artery disease (CAD) had significantly lower expression of circulating miRNAs compared with healthy individuals, and damaged endothelial cells can produce endothelial cell‐derived microparticles (EMP) with low miRNA expression 16. The PI3K/Akt/eNOS signalling pathway is closely related to apoptosis of endothelial cells, and Akt, a downstream effector of PI3K, activates eNOS, an important regulator of vascular wall homoeostasis, showing that the PI3K/Akt/eNOS signalling pathway plays a key role in the development of endothelial damage 14. eNOS can control the production of NO derived from endothelial cells to promote the migration and proliferation of endothelial cells, and the PI3K/Akt signalling pathway is involved in endothelial progenitor cell differentiation into endothelial cells (EPCs) 17. Additionally, abnormal NO signalling pathways can lead to vascular endothelial dysfunction, disruption of endothelial integrity and endothelial necrosis, all of which will give rise to inflammatory infiltration, plaque formation and thrombosis 18. NO is a bifunctional endothelial vasodilator that suppresses platelet aggregation, leucocyte adhesion and proliferation of vascular smooth muscle cells. However, excessive NO can react with superoxide anions and produce strong oxidizing peroxynitrite anions (ONOO‐), which can further result in lipid peroxidation and direct damage to endothelial cells 19. As shown in this study, compared with the blank group, PI3K, Akt and eNOS mRNA levels were up‐regulated in the miR‐138 inhibitor group but were down‐regulated in the miR‐138 mimic and LY294002 groups. The miR‐138 mimic and LY294002 groups had decreased cell proliferation and increased cell apoptosis, which suggested that up‐regulation of miR‐138 can alleviate HCAEC injury by inhibiting the PI3K/Akt/eNOS signalling pathway. Dasgupta *et al*. 20 showed that atherosclerosis is initiated and progresses through continuous injury of the vascular endothelial cells, resulting in the activation and apoptosis of endothelial cells. Liu *et al*. 21 also reported a negative association between miR‐138 and the PI3K/AKT signalling pathway, which together with our results demonstrated that this association can inhibit proliferation and promote apoptosis of endothelial cells.

We also found that compared to the blank group, the levels of TNF‐α, IL‐6, and IL‐8 were significantly decreased in the miR‐138 mimic and LY294002 groups but substantially increased in the miR‐138 inhibitor group, which indicated that up‐regulation of miR‐138 and inhibition of the PI3K/Akt/eNOS signalling pathway can suppress the inflammatory response in injured HCAECs. In addition, IL‐4 and IL‐10 were significantly increased in the miR‐138 mimic and LY294002 groups but decreased in the miR‐138 inhibitor group. Several reports have investigated the relationship between atherosclerosis and various factors, including endothelial cell injury, lipid infiltration, inflammatory response and thrombosis 22. To the best of our knowledge, HCAECs, in the normal state, can express 59 inflammatory genes, such as chemokines, cytokines, chemokine and cytokine receptors, and inflammatory transcription factors; thus, inflammatory cytokines will consequently be secreted by inflammatory activation of the HCAECs 23. The miR‐138 level is strongly associated with pro‐inflammatory cytokines, and if miR‐138 is suppressed, pro‐inflammatory signal transduction cascades will consequently be activated and maintained because of the restoration of eNOS activity, illustrating the possible relationship between miR‐138 and inflammatory activation of HCAECs 3, 24. However, previous studies have demonstrated that IL‐10 and IL‐4 were anti‐inflammatory cytokines 25, 26. As small bioactive proteins, cytokines regulate cellular growth, function and differentiation and control the immune response and inflammation, including Th1 cytokines, such as TNF‐α, and pro‐inflammatory cytokines, such as IL‐6 and IL‐8 27. As cellular responses, such as the pro‐inflammatory process, are involved in the production of cytokines, including IL‐6 and TNF‐α, and vascular complications will improve if the PI3K/Akt/eNOS pathway is suppressed, we have been suggested that these cytokines will decrease if the PI3K/Akt‐eNOS pathway is suppressed 17, 28.

In conclusion, this study indicates that up‐regulation of miR‐138 alleviates HCAEC injury and inflammatory response by inhibiting the PI3K/Akt/eNOS signalling pathway, which elucidates the underlying mechanism of HCAEC repair and further suggests therapeutic strategies to preserve endothelial integrity and vascular health and inhibit inflammatory activation in cardiovascular disease.

## Competing interests

None.
