# CORRIGENDUM

**DOI:** 10.1111/jcmm.15631

**Published:** 2020-09-27

In Li et al,^1^ the published article contains errors in Figures 2B and 4B. The correct figures are shown below. The authors confirm all results and conclusions of this article remain unchanged.

**FIGURE 2:**
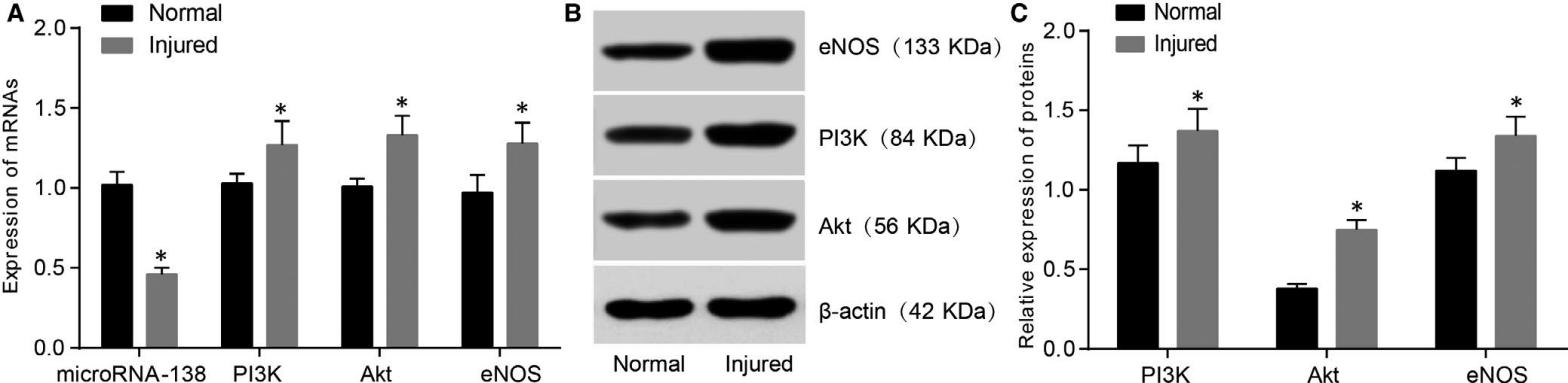
Comparisons of miR‐138, PI3K, Akt and eNOS expressions between the normal HCAECs and injured HCAECs. Note: A, qRT‐PCR was used to detect the expression of miR‐138 and the mRNA expressions of PI3K, Akt and eNOS in the normal HCAECs and injured HCAECs; B, Western blotting was used to detect the protein expressions of PI3K, Akt and eNOS in the normal HCAECs and injured HCAECs; C, PI3K, Akt and eNOS protein expressions; *P < .05 compared with the normal HCAECs; HCAECs, human coronary artery endothelial cells; qRT‐PCR, quantitative real‐time polymerase chain reaction; PI3K, phosphatidylinositol 3‐kinase; Akt, protein kinase B, eNOS, endothelial nitric oxide synthase

**FIGURE 4:**
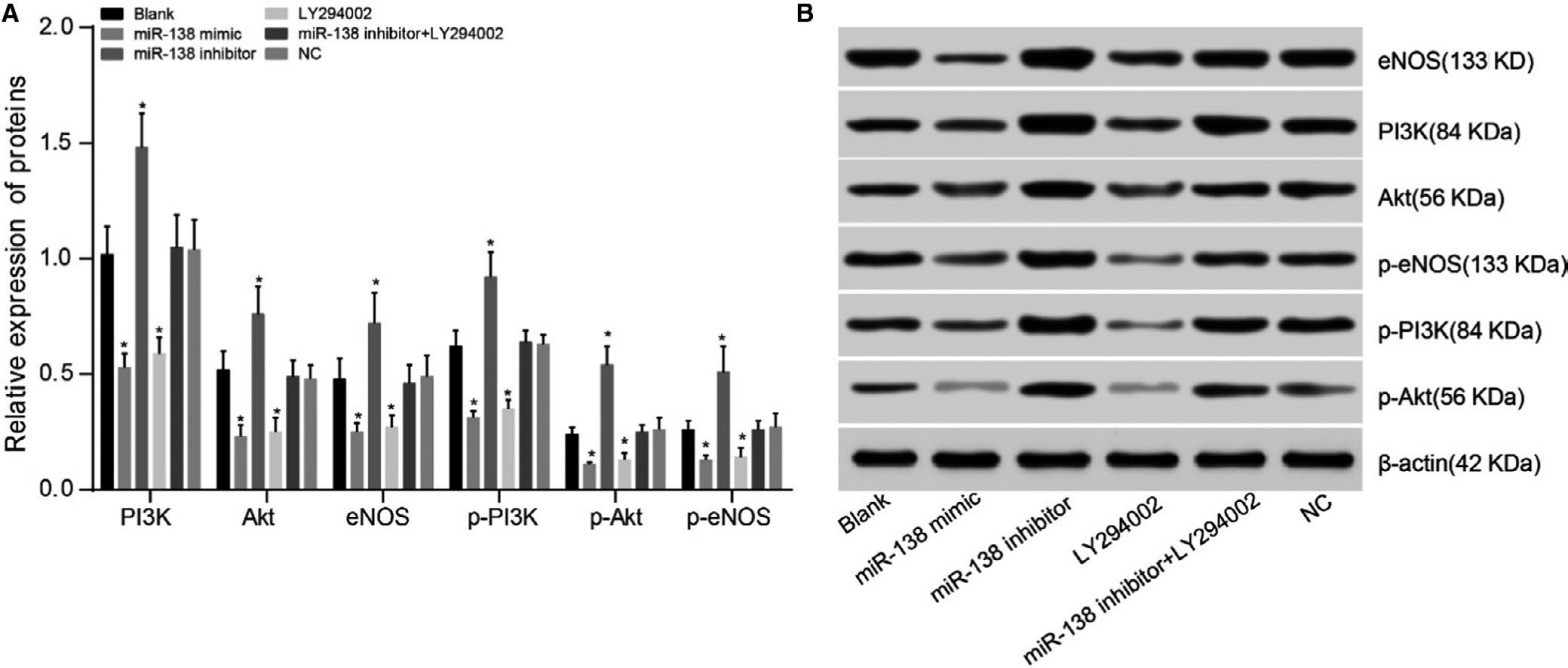
Comparisons of PI3K, Akt and eNOS protein expressions among five groups after transfection. Note: A, relative expressions of PI3K, Akt, eNOS, p‐PI3K, p‐Akt and p‐eNOS proteins; *P < .05 compared with the blank group; B, protein expressions of PI3K, Akt, eNOS, p‐PI3K, p‐Akt and p‐eNOS detected by Western blotting; PI3K, phosphatidylinositol 3‐kinase; Akt, protein kinase B, eNOS, endothelial nitric oxide synthase
